# Novel Repair Technique for Iatrogenic Femoral Artery Pseudoaneurysms Using a Suture-Mediated Closure Device Combined With Bidirectional Guidewire Crossing: A Case Report

**DOI:** 10.7759/cureus.79779

**Published:** 2025-02-27

**Authors:** Eiji Miyauchi, Syousei Osako, Ryo Arikawa, Naoya Oketani, Mitsuru Ohishi

**Affiliations:** 1 Department of Cardiology, Kagoshima City Hospital, Kagoshima, JPN; 2 Department of Cardiovascular Medicine and Hypertension, Graduate School of Medical and Dental Sciences, Kagoshima, JPN

**Keywords:** bidirectional approach, endovascular treatment (evt), iatrogenic complication, iatrogenic pseudoaneurysm, vascular closure device efficacy

## Abstract

A novel approach for iatrogenic femoral artery pseudoaneurysm (IFAP) using a suture-mediated closure device and bidirectional guidewire percutaneously and intravascularly will be presented in this case report. We report a case of a 73-year-old male patient who faced a complication due to an IFAP following a percutaneous coronary intervention. Manual compression and thrombin injection combined with intravascular balloon inflation were not effective. We then inserted a guidewire percutaneously to use a suture-mediated closure device, but it could not pass through the pseudoaneurysm neck into the common femoral artery. We then attempted a novel technique involving bidirectional guidewire crossing to use the suture-mediated closure device. First, two guidewires were inserted into the pseudoaneurysm cavity percutaneously and intravascularly. Second, the intravascular guidewire was replaced with a snare using a microcatheter to catch the percutaneous guidewire and pull it through the pseudoaneurysm neck into the femoral artery. Third, the percutaneous guidewire was replaced with a 0.035 guidewire to use the suture-mediated closure device. Finally, we confirmed that the IFAP disappeared angiographically. Our new approach could be an alternative method for treating difficult cases. Additional studies may be required to evaluate the long-term safety and efficacy of this approach.

## Introduction

Iatrogenic femoral artery pseudoaneurysm (IFAP) is a common complication in percutaneous endovascular procedures [1]. To address an IFAP, several methods are commonly used, such as manual compression and thrombin injection under ultrasound guidance, as well as combining these techniques with balloon inflation from within the vessel [2-4]. Recently, the use of a closure device for repairing an IFAP with a short and wide pseudoaneurysm neck has also been reported [5,6]. This method involves inserting a guidewire percutaneously through the pseudoaneurysm cavity and the neck, ultimately into the common femoral artery. However, the guidewire manipulation can be difficult when the pseudoaneurysm cavity is relatively small. In such cases, there have been reports that switching to a smaller-diameter guidewire can improve the likelihood of successful passage [7]. Nevertheless, if the guidewire still fails to pass, converting to surgical hemostasis becomes necessary. In this study, we present a case of IFAP using bidirectional guidewire crossing to enable the use of a suture-mediated closure device.

This study was previously presented as a meeting abstract at the 2024 ENCORE Seoul Annual Scientific Meeting on October 10, 2024.

## Case presentation

**Figure 1:**
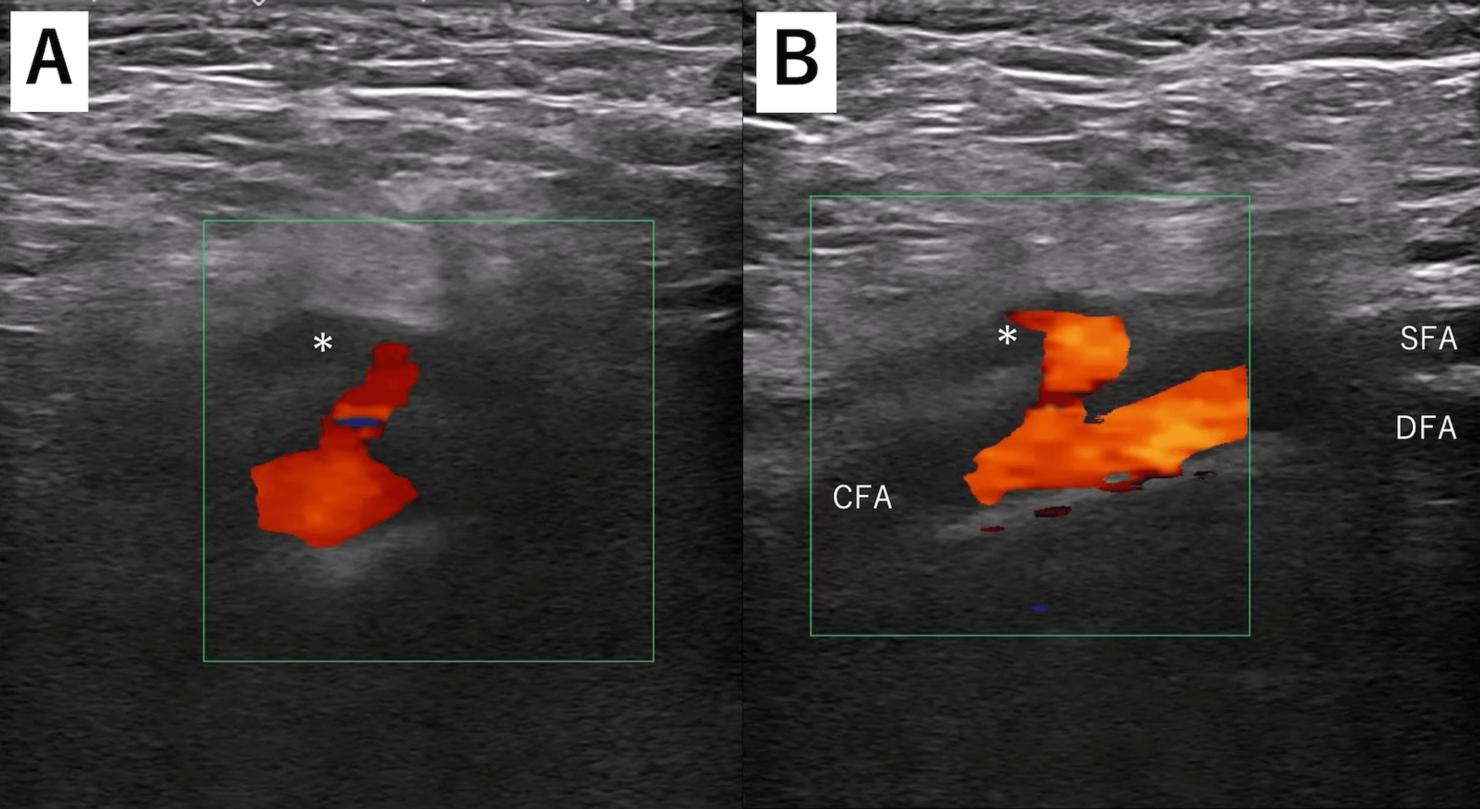
Color Doppler images of the pseudoaneurysm on the common femoral artery. Color Doppler images show a 9.3- × 3.3-mm pseudoaneurysm (asterisk) on the common femoral artery. (A) Short-axis view; (B) long-axis view. CFA: common femoral artery; SFA: superficial femoral artery; DFA: deep femoral artery

A 73-year-old male patient who had hypertension and diabetes and was receiving dialysis because of diabetic nephropathy was diagnosed with effort angina. He underwent percutaneous coronary intervention using a 7 French (Fr) sheath inserted in the right common femoral artery (CFA) because he got a functioning fistula on his left arm for dialysis, and we needed to keep his right radial artery patent in case his functioning fistula on his left arm would be occluded. Hemostasis at the puncture site was obtained with manual compression, but a palpable tumor at the puncture site was observed on the same day. A pseudoaneurysm beside the right femoral artery was detected by ultrasound imaging (Figures 1A, 1B).

**Figure 2:**
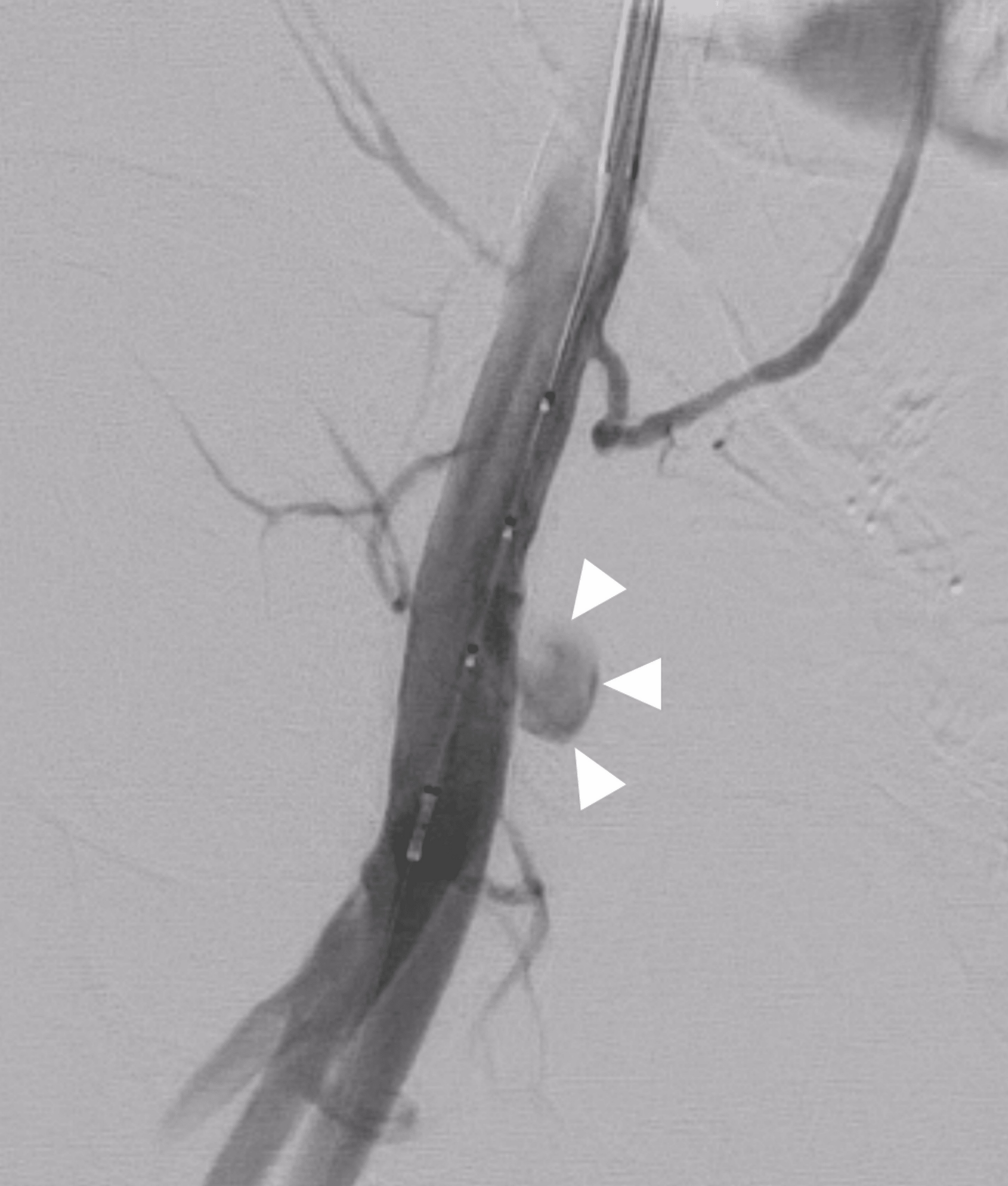
Angiography of the right femoral artery using a 6 French guiding sheath. A pseudoaneurysm cavity (white arrowheads) is seen arising from the right common femoral artery.

Fifteen-minute ultrasound-guided manual compression twice was not effective in repairing the pseudoaneurysm. We then inserted a guide sheath contralaterally via the left-to-right CFA to position it close to the aneurysm using contrast imaging (Figure 2).

**Figure 3:**
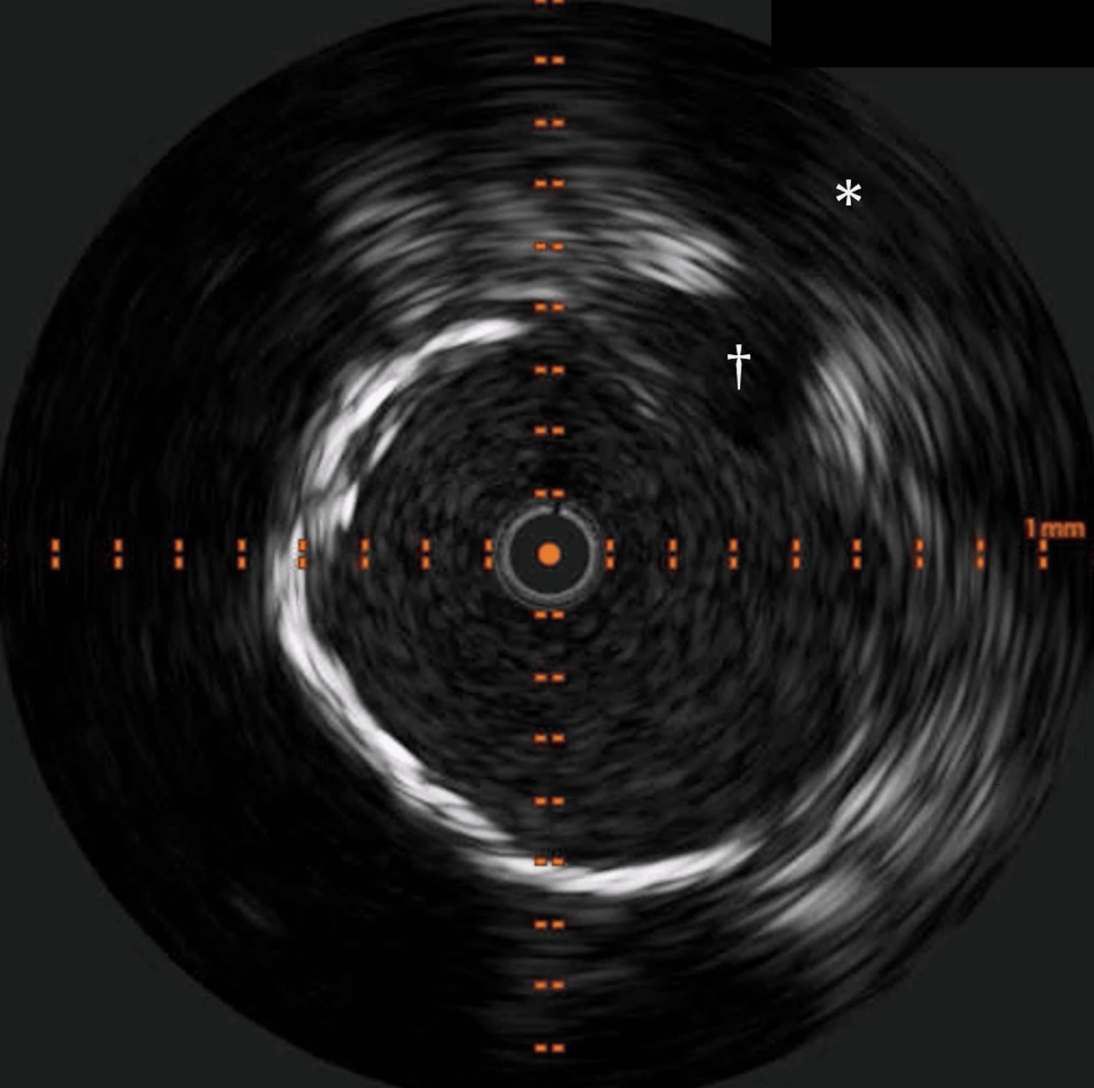
Intravascular ultrasound image at the pseudoaneurysm neck. The intravascular ultrasound image at the pseudoaneurysm neck (dagger) shows a neck width of approximately 2.7 mm. The lumen of the common femoral artery communicates with the cavity (asterisk) through the neck.

Subsequently, a balloon was inserted and inflated in the right CFA to interrupt blood flow to the pseudoaneurysm cavity to help repair the pseudoaneurysm with manual compression. Unfortunately, this strategy also failed to repair the pseudoaneurysm. We then injected 300 units of thrombin into the pseudoaneurysm cavity percutaneously using a 20-gauge needle combined with intravascular balloon inflation to interrupt blood flow to the cavity. However, this strategy also failed to repair the pseudoaneurysm; coagulation did not fully occur in the pseudoaneurysm with thrombin injection. Fortunately, distal embolization did not occur, though. Intravascular ultrasound showed that the diameter of the pseudoaneurysm neck was large (approximately 2.7 mm), while its length was short (approximately 3.0 mm). These features might be the main reason why the IFAP was not repaired by manual compression and/or thrombin injection (Figure 3).

**Figure 4:**
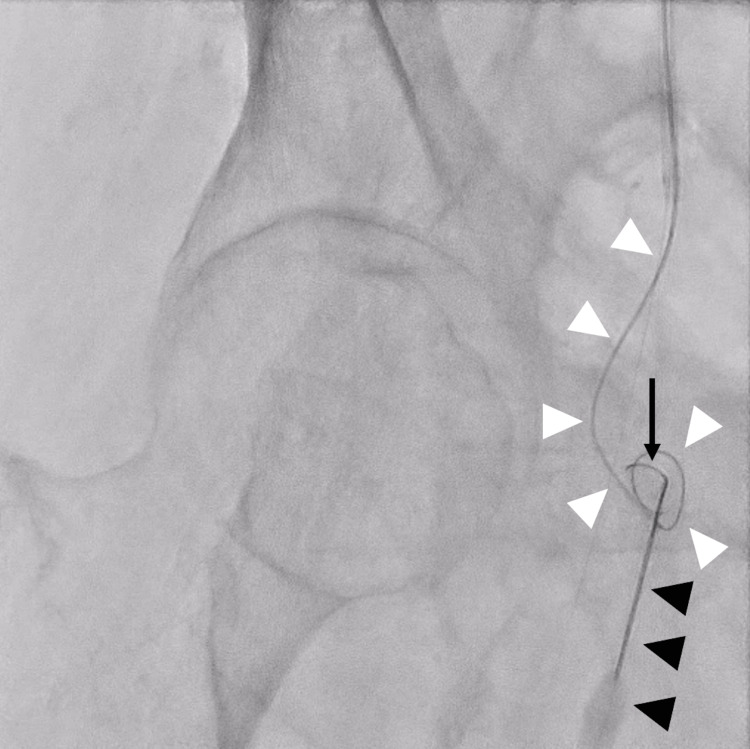
Fluoroscopic image show a guidewire inserted into the pseudoaneurysm cavity via a percutaneous needle. The fluoroscopic image shows a 0.014 guidewire (black arrow) inserted into the pseudoaneurysm cavity via a percutaneous needle (black arrowheads). An additional 0.014 guidewire with a microcatheter (white arrowheads) was inserted from the common femoral artery.

Following a discussion with vascular surgeons, we decided to use a suture-mediated closure device (Perclose Prostyle™; Abbott Vascular Inc., Santa Clara, CA, USA) to suture the neck of the IFAP using ultrasound guidance. We percutaneously punctured the pseudoaneurysm cavity from the center or opposite side of the pseudoaneurysm neck using surface ultrasound guidance with a 20-gauge needle and inserted a 0.014 nitinol guidewire with a hydrophilic coating (Jupitar FC, Boston Scientific, Marlborough, MA, USA). However, the puncture needle angle and pseudoaneurysm neck were not coaxial but were perpendicular instead. Therefore, the guidewire did not pass through the pseudoaneurysm neck to the femoral artery. A 0.014 guidewire was then inserted from the CFA from the contralateral sheath, and it proceeded to the pseudoaneurysm cavity through the neck (Figure 4).

**Figure 5:**
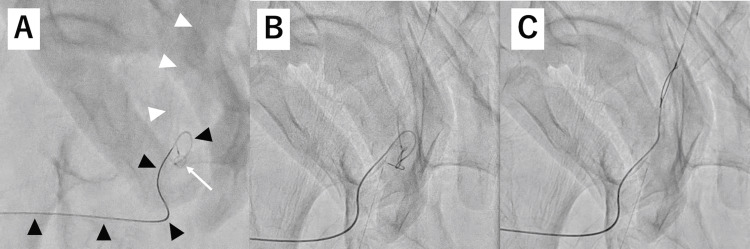
A series of fluoroscopic images show a percutaneously inserted 0.014 guidewire being pulled into the common femoral artery using a snare. (A) The microcatheter (black arrowheads) was percutaneously inserted, and the snare was inserted from the common femoral artery (white arrow). The microcatheter and the snare were seen negotiating with each other inside the pseudoaneurysm cavity. (B) The snare from the common femoral artery was grasping the 0.014 guidewire percutaneously inserted inside the pseudoaneurysm cavity. (C) The 0.014 guidewire was pulled out from the pseudoaneurysm cavity to the common femoral artery using the snare.

Therefore, using a microcatheter, we decided to replace the 0.014 guidewire inserted intravascularly with a 2 mm-diameter micro snare (Goose Neck™ Snare, Medtronic Japan, Tokyo, Japan) to grasp the 0.014 guidewire inserted percutaneously. After some attempts, we successfully grabbed the percutaneous guidewire with the snare and subsequently pulled it through the CFA (Figures 5A-5C, Video 1). 

**Video 1:** Intravascular snare grasping and pulling a percutaneously inserted guidewire in the pseudoaneurysm The percutaneously inserted guidewire was successfully grabbed with the intravascular snare and subsequently pulled through the common femoral artery from the pseudoaneurysm cavity.

**Figure 6:**
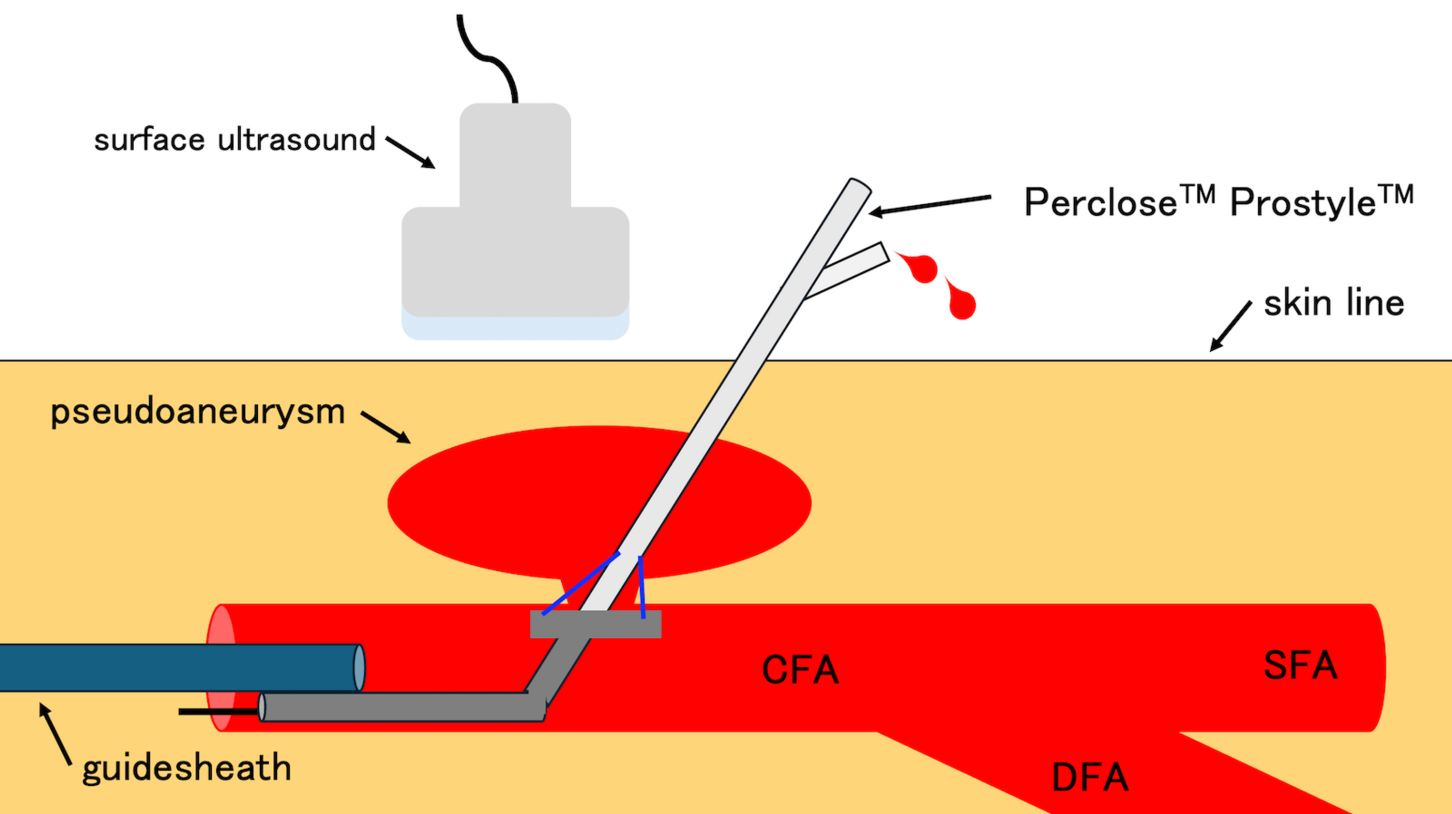
A hand-drawn diagram to illustrate the point of the confirmation of the foot position of the Perclose Prostyle using surface ultrasound. Using surface ultrasound, the Perclose Prostyle™ foot was pressed against the inside of the vessel wall rather than inside the pseudoaneurysm. CFA: common femoral artery; SFA: superficial femoral artery; DFA: deep femoral artery This image has been created by the authors.

Using this 0.014 guidewire, we inserted a 3 Fr sheath into the CFA. The 0.014 guidewire was then replaced with a 0.035 guidewire, which enabled the use of the Perclose Prostyle™. Using surface ultrasound, we confirmed that the foot of the Perclose Prostyle™, which was responsible for suturing, was in the right position (Figure 6).

**Figure 7:**
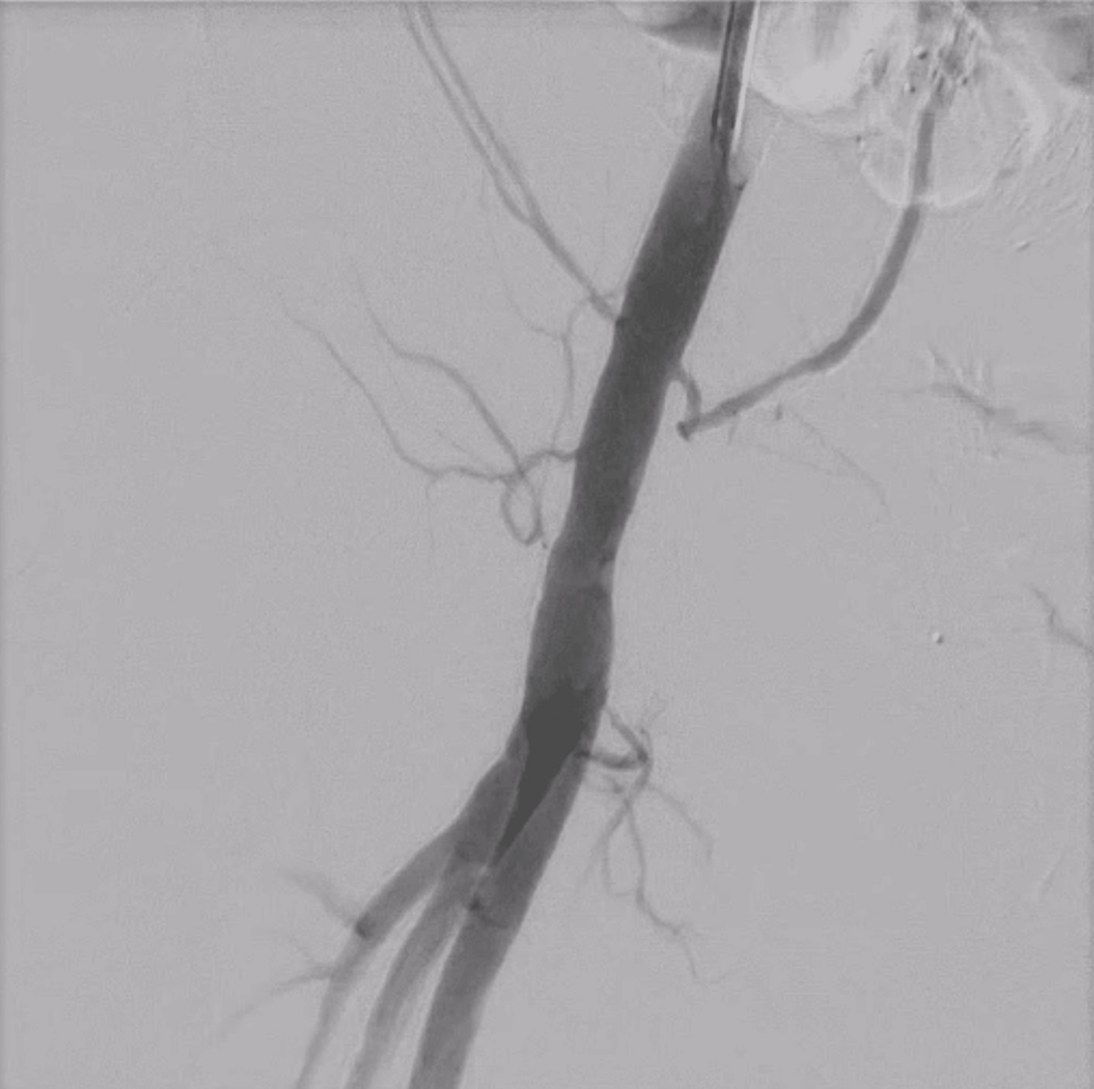
Angiographic image of the right common femoral artery after suturing the pseudoaneurysm neck using the Perclose Prostyle. No contrast agent flow was detected into the cavity.

The Perclose Prostyle™ was then used to suture the vascular wall beside the pseudoaneurysm. After this procedure, no contrast proceeded to the pseudoaneurysm cavity angiographically (Figure 7).

We also achieved hemostasis in the contralateral common femoral artery, which served as the sheath insertion site, using the Perclose Prostyle™. The day after the procedure, dual antiplatelet therapy and dialysis using heparin were restored. There were no additional perioperative complications, and the patient was discharged two days after this procedure, with an uneventful course thereafter.

## Discussion

An IFAP is one of the most common complications in percutaneous endovascular procedures [5]. An IFAP is usually successfully repaired with ultrasound-guided manual compression, thrombin injection, or combining these techniques with intravascular balloon inflation [2-4]. Recent reports have described using the Perclose Prostyle™ to repair IFAPs with short, wide-neck formations [5]. In the previously described cases, a guidewire percutaneously travels through the neck of the pseudoaneurysm to the femoral artery, with guidance using ultrasound or contrast [5,8,9]; however, this was not possible in our case. Manipulation of the guidewire is difficult when the puncture needle angle and pseudoaneurysm neck are not coaxial. Therefore, a snare deployed from the contralateral femoral puncture was used to catch the percutaneous wire in our case. We inserted a guidewire intravascularly into the pseudoaneurysm cavity, replaced it with a small snare, grasped the 0.014 guidewire inserted percutaneously, and finally pulled it into the common femoral artery. This allowed us to insert a 3 Fr sheath and close the neck with the Perclose Prostyle™. Using this technique, surgeons need to understand the anatomical position between the CFA, pseudoaneurysm neck, and cavity, using imaging modalities such as angiography, intravascular ultrasound, and surface ultrasound. The use of microcatheters or shaped-tip angiographic catheters also facilitates guidewire manipulation.

When using the Perclose Prostyle™, there are two important points to consider. One consideration is the neck diameter. Generally, a single Perclose Prostyle™ device safely closes a vessel up to approximately 3.0 mm. An accurate assessment of the neck diameter with intravascular or surface ultrasound is crucial before performing this technique. When the neck diameter is more than 3.0 mm, using dual Perclose Prostyle™ devices for percutaneous suturing or surgical repair needs to be considered [10,11]. The second consideration is confirming the foot positioning of the device. Unlike in normal vessel closure with the Perclose Prostyle, the foot position of the device cannot be verified by blood backflow through the marker tube. Therefore, surface ultrasound should be used to ensure the proper foot position. Our IFAP repair technique featuring a bidirectional approach and the use of a snare may effectively repair IFAPs with wide necks and small cavities, which may not be treatable with conventional techniques. Further accumulation of cases is required to evaluate the long-term safety and effectiveness of this technique, define its indications, and examine potential applications in other vascular regions.

## Conclusions

Our technique, combining a bidirectional approach with a suture-mediated closure device, enables successful IFAP closure in cases where conventional methods are unsuitable. Our technique is particularly useful for pseudoaneurysms with a wide neck and small cavity. Further studies are required to assess the long-term safety and success to clarify the indications for this method.

## References

[1] Schneider C, Malisius R, Küchler R, Lampe F, Krause K, Bahlmann E, Kuck KH (2009). A prospective study on ultrasound-guided percutaneous thrombin injection for treatment of iatrogenic post-catheterisation femoral pseudoaneurysms. Int J Cardiol.

[2] Coley BD, Roberts AC, Fellmeth BD, Valji K, Bookstein JJ, Hye RJ (1995). Postangiographic femoral artery pseudoaneurysms: further experience with US-guided compression repair. Radiology.

[3] Chen DH, Sammel AM, Jain P, Jepson NS (2015). Cardiologist operated ultrasound guided thrombin injection as a safe and efficacious first line treatment for iatrogenic femoral artery pseudoaneurysms. Heart Lung Circ.

[4] Hayakawa N, Kodera S, Miyauchi A (2022). Effective treatment of iatrogenic femoral pseudoaneurysms by combined endovascular balloon inflation and percutaneous thrombin injection. Cardiovasc Interv Ther.

[5] Kodama T, Yamaguchi T, Fujiwara H, Kuwabara M (2022). Successful endovascular repair of complicated pseudoaneurysm using Perclose ProGlide: a novel concept. Clin Case Rep.

[6] Hadziomerovic A, Jetty P, Gupta A (2016). Angioseal-assisted closure of iatrogenic refractory femoral arterial pseudoaneurysm: a novel technique. JACC Cardiovasc Interv.

[7] Inagaki Y, Nakao M, Arashi H, Yamaguchi J (2022). Novel interventional technique for the treatment of an iatrogenic pseudoaneurysm of the brachial artery. J Cardiol Cases.

[8] Gong X, Zhang W, Sang L, Sun Y, Yu M (2021). Successful treatment of a femoral pseudoaneurysm by ultrasonographically-guided application of a suture-mediated closure device. J Clin Ultrasound.

[9] Liu W, Liu C, Lu SY (2020). Percutaneous suture technique with ProGlide to manage vascular access pseudoaneurysm after percutaneous coronary intervention procedure: a case report. Chin J Traumatol.

[10] Maeno R, Taniguchi R, Suhara M, Mochizuki Y, Takayama T, Hoshina K (2023). Area reduction of perforation with a small-size sheath technique for iatrogenic femoral artery pseudoaneurysm with a large perforation. J Vasc Surg Cases Innov Tech.

[11] Wu H, Zhang L, Zhang C, Xie B, Lou C, Liu Y, Bai H (2022). Non-surgical treatment versus surgery for iatrogenic femoral artery pseudoaneurysms: systematic review and meta-analysis. Front Surg.

